# Tregs and GvHD prevention by extracorporeal photopheresis: observations from a clinical trial

**DOI:** 10.1186/s40164-021-00210-9

**Published:** 2021-02-16

**Authors:** Crocchiolo Roberto, Cesana Clara, Girgenti Debora, Bertani Giambattista, Barba Claudia, Liga Giuseppa, Ferri Ursula, Crucitti Lara, Grillo Giovanni, Rossini Silvano, Cairoli Roberto

**Affiliations:** 1 Servizio Di Immunoematologia E Medicina Trasfusionale, ASST Grande Ospedale Metropolitano Niguarda, Piazza Dell’Ospedale Maggiore, 3, 20162 Milano, Italy; 2 Ematologia, ASST Grande Ospedale Metropolitano Niguarda, Milano, Italy

**Keywords:** Graft-versus-host disease, T regulatory cells, Extracorporeal photopheresis, Allogeneic hematopoietic stem cell transplantation

## Abstract

The aim of the present study was to evaluate the circulating T regulatory cells (Tregs) in patients undergoing extracorporeal photopheresis (ECP) for the prevention of chronic graft-versus-host disease (GvHD) and to search for any correlation between Tregs counts and chronic GvHD occurrence. Among n = 12 patients with complete longitudinal data, the median cumulative values of absolute peripheral Tregs counts were 21.64 and 63.49 cells/µL for patients who developed chronic GvHD and those who did not develop it, respectively (p = 0.05). The analysis of the median absolute counts of peripheral HLA-DR + Tregs provided similar results, showing that 20% (1 out of 5) and 100% (7 out of 7) of patients with HLA-DR + Tregs values of > 5 cells/µL were in the GvHD and non-GvHD groups, respectively (p = 0.01). In conclusion, the present results support the involvement of Tregs in the prevention of chronic GvHD in patients receiving ECP and suggest Tregs count as a potential biomarker of ECP effectiveness. Future strategies are needed to enhance Tregs expansion and/or activity in conjunction with ECP for an effective chronic GvHD prevention.

To the Editor,

Graft-versus-host disease (GvHD) represents a life-threatening complication of allogeneic hematopoietic stem cell transplantation (HSCT), causing significant morbidity and mortality after transplant. The immunotolerant CD4 + /CD25 + /FOXP3 + lymphocyte subset, namely T regulatory cells (Tregs), is recognized for having an important role during GvHD [1], and its expansion following sirolimus administration contributes to an effective GvHD prevention [2]. Extracorporeal photopheresis (ECP), a well-known treatment for both acute and chronic GvHD [3] acts through an immunomodulatory action on different components of the immune system, included the Tregs compartment; however, data on the effect played by ECP on Treg population are conflicting because some studies report expansion associated with response [4, 5] while others do not [6, 7].

Here we present ex-vivo data from a clinical trial of ECP administered for GvHD prevention, suggesting a role of Tregs on chronic GvHD occurrence. The trial (Eudract 2008–007,108-27) enrolled n = 18 patients undergoing HSCT who received ECP starting from month 6 after HSCT up to month 18 and until GvHD occurrence or relapse, once a week in months 6–7, then every two weeks in months 8–9 and once a month in months 10 to 18. Our report focuses on n = 12 patients for whom sequential Tregs counts during the first month of ECP were available, weekly from the date of ECP start. Peripheral blood samples were taken before each ECP procedure. Cumulative values of absolute peripheral Tregs and of peripheral HLA-DR + Tregs counts were obtained by the sum of the four weekly results, for each patient; then, median values were compared after grouping patients into those subsequently developing (or not) GvHD. Main patients´ characteristics and ECP duration are shown in Table 1. Treg cells were identified on flow cytometry by gating the CD4 + /CD25 + /CD127− population, further divided into HLA-DR + and HLA-DR− after selection of CD39 + /CD45RA− lymphocytes.

**Table 1:** Patients characteristics at allogeneic HSCT

Median age, years (range)	62 (36–67)
Gender M/F, number (%)	5 (42)/7 (58)
Diagnosis, number (%)	
Acute Myeloid Leukemia	6 (50)
Non-Hodgkin´s Lymphoma	3 (25)
Myelodysplastic Syndrome	2 (17)
Hodgkin´s Lymphoma	1 (8)
Disease status at HSCT, number (%)	
CR1	4 (33)
CR2	6 (50)
PR	2 (17)
Hematopoietic stem cell donor, number (%)	
HLA-identical sibling	6 (50)
Unrelated donor	6 (50)
Median CD34 + infused, 10^6^/kg (range)	6.47 (4.90–10.41)
Median CD3 + infused, 10^8^/kg (range)	9.92 (6.23–19.61)
Median ECP duration, months (range)	5 (1–12)

HSCT, hematopoietic stem cell transplantation; M, male; F, female; CR1, first complete remission; CR2, second complete remission; PR, partial remission

Interestingly, we found that the n = 5 patients who further developed chronic GvHD had significant lower levels of circulating Tregs compared with the remaining n = 7 patients, not developing GvHD. Indeed, the cumulative values of absolute peripheral Tregs counts, expressed in cells/µL, were 9.14, 12.50, 21.64, 46.85, 68.37 and 36.68, 38.43, 44.37, 63.49, 75.04, 82.02, 94.81 in the GvHD and non-GvHD patients, respectively, with a median of 21.64 vs. 63.49 cells/µL (p = 0.05, Mann–Whitney test, one-sided). The analysis of the median absolute counts of peripheral HLA-DR + Tregs provided similar results (Fig. 1).

**Fig. 1:**
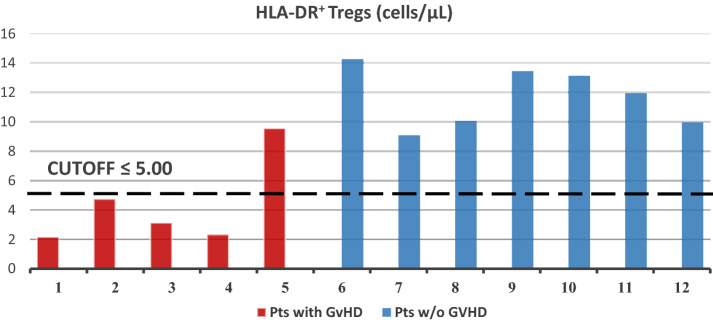
Analysis of the median absolute counts of peripheral HLA-DR + Tregs in patients with and without GvHD. Data show that 20% (1 out of 5) vs. 100% (7 out of 7) of patients with HLA-DR + Tregs had values of > 5 cells/µL in the GvHD vs. non-GvHD groups, respectively (p = 0.01, Fisher exact test)

The present results support the involvement of Tregs in the prevention of chronic GvHD in patients receiving ECP. Although the observation is based on low numbers and the results derive from post-hoc analyses, our data confirm the importance of this cellular subset as an immunotolerant player during inflammation and alloreactivity in the post-HSCT period [8, 9]. The immunological mechanisms by which ECP exerts its action on GvHD are not fully elucidated; nonetheless, the modulation of some cellular subsets like circulating dendritic cells, Treg population or the shift of pro- and anti-inflammatory cytokine profiles are well-documented and may explain the clinical benefit observed in most patients with GvHD [10]. Our findings support the hypothesis of a protective role played by Tregs and link this cellular subset to ECP effectiveness; moreover, the Tregs absolute count here might represent a biomarker of activity and this is in line with what observed by Machado Lopes et al. [11] in the therapeutic setting, where the current lack of reproducible biomarkers is an unmet clinical need to be fulfilled, hopefully in the near future. Notably, here ECP was administered as a preventative measure with the aim of avoiding chronic GvHD, conversely to the majority of published data that refer to the treatment of GvHD, once it occurred (to our knowledge, the only study of prophylactic ECP [12] did not investigate the Treg compartment); our data thus demonstrate that the Tregs hypothesis may also apply to the prophylactic setting, although it has to be confirmed by further studies. In addition, Tregs might act as a biomarker of ECP effectiveness in preventing GvHD, deserving to be addressed in future studies.

In conclusion, taking into account the data from us and other groups, we believe that future strategies are needed to enhance Tregs expansion and/or activity in conjunction with ECP for an effective GvHD prophylaxis after HSCT.

## Data Availability

The datasets used and/or analysed during the current study are available from the corresponding author on reasonable request.

## Abbreviations

GvHD: Graft-versus-host disease
HSCT: Hematopoietic stem cell transplantation
Tregs: T regulatory cells
ECP: Extracorporeal photopheresis
