# Correction to: Tregs and GvHD prevention by extracorporeal photopheresis: observations from a clinical trial

**DOI:** 10.1186/s40164-021-00216-3

**Published:** 2021-03-17

**Authors:** Roberto Crocchiolo, Clara Cesana, Debora Girgenti, Giambattista Bertani, Claudia Barba, Giuseppa Liga, Ursula Ferri, Lara Crucitti, Giovanni Grillo, Silvano Rossini, Roberto Cairoli

**Affiliations:** 1 Servizio Di Immunoematologia E Medicina Trasfusionale, ASST Grande Ospedale Metropolitano Niguarda, Piazza Dell’Ospedale Maggiore, 3, 20162 Milano, Italy; 2 ASST Grande Ospedale Metropolitano Niguarda, Milano, Italy

## Correction to: Exp Hematol Oncol (2021) 10:14 https://doi.org/10.1186/s40164-021–00210-9

Following publication of the original article [1], the authors reported error in author name. The authors first name and surnames are swapped in the submitted version and published.

The original article has been corrected.
